# Initial distribution volume of glucose can be approximated using a conventional glucose analyzer in the intensive care unit

**DOI:** 10.1186/cc3047

**Published:** 2005-02-11

**Authors:** Hironori Ishihara, Hitomi Nakamura, Hirobumi Okawa, Hajime Takase, Toshihito Tsubo, Kazuyoshi Hirota

**Affiliations:** 1 Department of Anesthesiology, University of Hirosaki School of Medicine, Hirosaki-Shi, Japan; 2 Department of Anesthesiology, University of Hirosaki School of Medicine, Hirosaki-Shi, Japan; 3 Intensive Care Unit, University of Hirosaki Hospital, Hirosaki-Shi, Japan; 4 Department of Anesthesiology, University of Hirosaki School of Medicine, Hirosaki-Shi, Japan; 5 Intensive Care Unit, University of Hirosaki Hospital, Hirosaki-Shi, Japan; 6 Department of Anesthesiology, University of Hirosaki School of Medicine, Hirosaki-Shi, Japan

**Keywords:** distribution volume, glucose, measurement techniques, plasma, whole blood

## Abstract

**Introduction:**

We previously reported that initial distribution volume of glucose (IDVG) reflects central extracellular fluid volume, and that IDVG may represent an indirect measure of cardiac preload that is independent of the plasma glucose values present before glucose injection or infusion of insulin and/or vasoactive drugs. The original IDVG measurement requires an accurate glucose analyzer and repeated arterial blood sampling over a period of 7 min after glucose injection. The purpose of the present study was to compare approximated IDVG, derived from just two blood samples, versus original IDVG, and to test whether approximated IDVG is an acceptable alternative measure of IDVG in the intensive care unit.

**Methods:**

A total of 50 consecutive intensive care unit patients were included, and the first IDVG determination in each patient was analyzed. Glucose (5 g) was injected through the central venous line to calculate IDVG. Original IDVG was calculated using a one-compartment model from serial incremental arterial plasma glucose concentrations above preinjection using a reference glucose analyzer. Approximated IDVG was calculated from glucose concentrations in both plasma and whole blood, using a combined blood gas and glucose analyzer, drawn at two time points: immediately before glucose injection and 3 min after injection. Subsequently, each approximated IDVG was calculated using a formula we proposed previously.

**Results:**

The difference (mean ± standard deviation) between approximated IDVG calculated from plasma samples and original IDVG was -0.05 ± 0.54 l, and the difference between approximated IDVG calculated from whole blood samples and original IDVG was -0.04 ± 0.61 l. There was a linear correlation between approximated and original IDVG (r^2 ^= 0.92 for plasma samples, and r^2 ^= 0.89 for whole blood samples).

**Conclusion:**

Our findings demonstrate that there was good correlation between each approximated IDVG and original IDVG, although the two measures are not interchangeable. This suggests that approximated IDVG is clinically acceptable as an alternative calculation of IDVG, although approximated and original IDVGs are not equivalent; plasma rather than whole blood measurements are preferable.

## Introduction

We previously proposed initial distribution volume of glucose (IDVG), determined using injection of a small amount of glucose (5 g), as a measure of central extracellular fluid volume status [1-3]. Neither the plasma glucose values present before glucose injection nor infusion of insulin and/or vasoactive drugs had any apparent effect on IDVG calculation [1-3]. IDVG has been demonstrated to correlate well with cardiac output in various critically ill conditions in the absence of congestive heart failure [1,4]. We [5] and Gabbanelli and coworkers [6] recently showed that IDVG, rather than cardiac filling pressures, is clinically relevant as an indirect measure of cardiac preload, based on the close correlation between IDVG and intrathoracic blood volume, even though glucose administered intravenously distributes rapidly not only through the intravascular compartment but also through the extravascular space. Measurement of IDVG can be repeated at 30 min intervals [7,8]. Our original method for IDVG measurement requires repeated arterial blood samplings over 7 min after glucose injection. However, we have proposed that IDVG may be approximated using just two plasma samples, drawn immediately before injection and 3 min after injection [9]. In this manner, IDVG could be simply and rapidly assessed in the intensive care unit (ICU) if an accurate glucose analyzer were readily available.

Rapid and relatively accurate blood glucose measurement has become possible using combined blood gas and glucose analyzers. Many ICUs have this type of glucose analyzer, which would permit routine use of approximated IDVG as a measure of fluid volume in those units, provided that plasma or whole blood glucose concentrations measured using these devices are suitable for IDVG determination.

In the present study we compared approximated IDVG (calculated from plasma or whole blood samples using a combined blood gas and glucose analyzer) with original IDVG (measured using a laboratory reference method), and examined whether approximated IDVG is a clinically acceptable alternative measure of IDVG.

## Methods

The research protocol was approved by the Ethics Committee of the University of Hirosaki. Patients or their relatives gave informed consent. A total of 50 patients admitted to the general ICU of the University of Hirosaki Hospital between July and September 2004 were included in this prospective study (Table 1). Although patients may undergo several fluid volume determinations during their stay in the ICU, the present study considered only the first IDVG measurement in each patient during their stay in the ICU. We included 40 surgical patients who had undergone cardiac surgery, mostly coronary artery bypass grafting and aortic arch replacement (*n *= 23), major abdominal surgery such as bowel resection and oesophagectomy (*n *= 5), laryngectomy (*n *= 4), hip joint surgery (*n *= 4), thoracic surgery (*n *= 2), large vessel surgery (*n *= 1), or spine surgery (*n *= 1). The remaining 10 patients had nonsurgical pathology such as cardiac failure (*n *= 2), respiratory failure (*n *= 2), chest trauma (*n *= 2), renal failure (*n *= 1), water intoxication (*n *= 1), tetanus (*n *= 1) and heat stroke (*n *= 1).

To calculate IDVG 10 ml of 50% glucose solution (5 g) was injected through the central venous line, as reported previously [1-3]. Blood samples were obtained through a radial artery catheter immediately before and 3, 4, 5 and 7 min after injection. Each 2 ml blood sample was collected in a heparinized syringe. Both plasma and whole blood glucose concentrations were measured using a combined blood gas and glucose analyzer (EML100 Electrolyte Metabolite Laboratory; Radiometer, Copenhagen, Denmark) from two blood samples: one drawn immediately before glucose injection and one 3 min after injection. Other than automatic regular calibration, the analyzer was not calibrated. Plasma glucose concentrations in all blood samples were also measured using amperometry by glucose oxidase immobilized membrane–H_2_O_2 _electrode (glucose analyzer GA-1150; Arkray Co., Ltd, Kyoto, Japan) as the reference. The interassay coefficients of variation were 2.6% for the former and 0.3% for the latter at a glucose concentration of 150 mg/100 ml (*n *= 6). Original IDVG (the reference) was calculated from the plasma decay curve with a one-compartment model from plasma values increased above preinjection levels between 3 and 7 min postinjection, as described in our previous reports [1-5]. Akaike's information criterion (AIC) [10] for the original IDVG curve was examined, as described previously [1-5], to evaluate the exponential term of the pharmacokinetic model. The lower the AIC value, the better the fit between observed data and the plasma glucose decay curve.

Approximated IDVG was calculated from the increase in either plasma or whole blood glucose concentration above the preinjection level at 3 min after glucose injection using a combined blood gas and glucose analyzer, as described above. In addition, we calculated approximated IDVG from the increase in plasma values above baseline at 3 min after glucose injection determined using the reference glucose analyzer Each approximated IDVG was calculated according to the following formula (proposed by us [9]; Table 2): approximated IDVG (l) = 24.4 × exp^(-0.03 × Δgl) ^+ 2.7. (Δgl is the increase in glucose concentration above the preinjection level at 3 min after injection.)

Data are expressed as mean ± standard deviation (SD). Bland–Altman plots were used to compare the bias (the mean of the differences) and precision (SD of bias) between measurements. In addition, regression analysis or a t-test was performed in the comparison of two paired variables. *P *< 0.05 was considered statistically significant.

## Results

Glucose concentrations and other variables for approximated IDVGs are summarized in Table 3. Glucose concentrations in plasma were higher than in whole blood by an average of 2 ± 3 mg/100 ml (*n *= 100; *P *< 0.001). The mean haematocrit was 30.3 ± 5.5%, and the total plasma protein concentration was 5.1 ± 0.7 g/100 ml. Neither haematocrit nor total plasma protein concentration were correlated with differences in glucose values between plasma and whole blood samples.

Because the AIC value for original IDVG was -24.8 ± 5.5, convergence was assumed in each glucose decay curve in the present study, as was observed in previous reports [1-5]. The mean original IDVG was 7.44 ± 1.83 l and the rate of disappearance of glucose from plasma was 0.069 ± 0.018 min.

**Figure 1:**
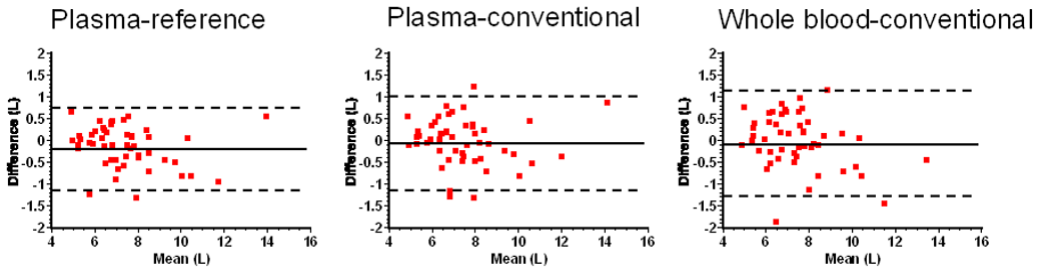
Bland–Altman plots of the differences between each approximated IDVG and original IDVG. Approximated IDVG was calculated from a formula using the increased glucose concentration above baseline at 3 min after injection of glucose [9]. Shown are the reference plasma glucose measurement (left), a conventional plasma glucose measurement (middle) and a conventional whole blood measurement (right). Solid lines represent the mean difference, and dashed lines represent the 95% confidence interval.

Bland–Altman plots of the differences between each approximated IDVG and original IDVG are shown in Fig. 1. There was a close correlation between each approximated IDVG and original IDVG (reference plasma values: *n *= 50, r^2 ^= 0.94, *P *< 0.0001; plasma values from the combined blood gas and glucose analyzer: *n *= 50, r^2 ^= 0.92, *P *< 0.0001; whole blood values from the combined blood gas and glucose analyzer: *n *= 50, r^2 ^= 0.89, *P *< 0.0001).

## Discussion

Although bedside reflectance glucometers rarely overestimate or underestimate the 'true' glucose concentration by more than 40 mg/100 ml (2.2 mmol/l) [11], this margin of error is too great for measurement of IDVG. In addition, plasma protein concentrations, haematocrit and body temperature, as well as blood oxygen tension, may influence measurements from such devices significantly [12-14]. Accordingly, bedside glucometers were not used in our measurement of IDVG. Instead, we used a conventional but more accurate glucose analyzer, specifically a combined blood gas and glucose analyzer.

We demonstrated that approximated IDVG, calculated from either plasma or whole blood values using a conventional glucose analyzer, is not markedly different from original IDVG, with the two measures correlating closely. We recently reported that repeated IDVG measurements, done at an interval of 30 min, differ by 0.08 ± 0.32 l in haemodynamically stable patients [8]. Based on this finding the limits of clinical agreement for IDVG measurement can be set at ± 0.4 l, although the limits within which the two methods were considered to be interchangeable were set at ± 0.5 l/min for measurement of cardiac output [15]. Although the difference between approximated and original IDVG in the present study was not particularly great, it extended beyond the limits of agreement. Our previous study [9] also showed that the difference between approximated and original IDVG was 0.03 ± 0.43 l in 150 paired data using the same reference plasma glucose measurement system, again indicating that the methods are not interchangeable. However, bearing in mind the close correlation between the two measures and the clinically applicable procedure for measurement of approximated IDVG, the latter – measured using a conventional glucose analyzer (but not a bedside reflectance glucometer) – may be useful in the ICU.

We previously proposed [1-3] that IDVG represents central extracellular fluid volume status, including plasma volume and the interstitial fluid volume of highly perfused organs such as brain, heart, lungs, liver and kidneys, without modification of glucose metabolism and regardless of the presence or absence of peripheral oedema. Glucose rapidly traverses the red cell membrane by facilitated diffusion without requiring energy or insulin [16]. Because the mass concentration of water in plasma is 0.93 kg H_2_O/l and that in red cells is 0.71 kg H_2_O/l, whole blood has a mass concentration of water of approximately 0.84 kg H_2_O/l. Although the molality of glucose in plasma (mmol/kg H_2_O) is equal in red cells, the glucose concentration in plasma (mmol/l) is greater than in either red cells or whole blood, depending on the haematocrit of the blood sample [16]. There was no significant correlation between haematocrit and the difference between paired plasma and whole blood glucose data in the present study (r^2 ^= 0.004), but the plasma glucose value was significantly greater than that in whole blood. However, the impact of this difference on incremental values would be less significant than that on absolute values. Thus, we may approximate IDVG from two whole blood glucose measurements, even measurements determined using a conventional glucose analyzer (but not a bedside reflectance glucometer). However, we believe that plasma glucose measurement is superior to whole blood glucose measurement, based on the bias and precision of the present data as well as by recommendation of plasma glucose rather than whole blood measurement, since the former is routinely used as the reference method [17].

Furthermore, a 5–10% decrease in whole blood glucose concentrations was observed during the first hour after sampling in routine conditions [18]. Whatever the calculation, it is important that all procedures be performed using proper technique and with an accurate sampling time.

The turnaround time for approximated IDVG measurement from the first blood sample to completion of the calculation is about 5 min in our ICU. In our experience, gained in more than 3500 determinations of original IDVG, it can be measured during routine fluid management, and it is not necessary to stabilize plasma glucose concentrations, provided that the infusion rate of glucose for routine fluid management remains unchanged before and during the measurement procedure. We observed a continuous decline in plasma glucose concentration over 60 min after injection, although plasma glucose concentrations at 60 min postinjection remained slightly elevated as compared with the preinjection value [8]. Hence, IDVG measurement will not induce a continued hyperglycaemic state, even in critically ill patients. However, Diaz-Parejo and coworkers [19] suggested that transient moderate hyperglycaemia had no adverse effect on outcome in patients with severe traumatic brain lesions and stroke. Therefore, we should be more concerned about normalization in basal plasma glucose concentration than about transient hyperglycaemia in these patients.

Gabbanelli and coworkers [6] utilized plasma glucose values, measured using a glucose analyzer similar to that used in the present study, to approximate IDVG based on the formula we proposed [9]. In accordance with our findings and corroborating our previous suggestions [1,3-5], those investigators found that approximated IDVG correlated well with both cardiac output and intrathoracic blood volume. Accordingly, either original or approximated IDVG is useful as an indirect measure of cardiac preload. Based on our clinical experience, normal IDVG is approximately 120 ml/kg, apparently high IDVG is above 140 ml/kg and apparently low IDVG is less than 100 ml/kg in the presence or absence of cardiac pathology or peripheral oedema. However, further detailed studies are required to determine the IDVG that are critical in terms of decision making regarding fluid management in different underlying pathologies.

## Conclusion

We calculated approximated IDVG from plasma and whole blood glucose concentrations measured using a combined blood gas and glucose analyzer. The results indicate that either calculation of approximated IDVG exhibits a close linear correlation with original IDVG measured using a reference glucose analyzer, although they are not interchangeable. Our findings suggest that approximated IDVG is clinically relevant because it may be used for point-of-care testing to assess fluid volume.

## Key messages

• 	IDVG has been proposed to be an indirect measure of cardiac preload without significant modification of glucose metabolism, but requiring repeated arterial blood samplings over 7 min after injection of glucose 5 g.

• 	Approximated IDVG derived from just two blood samples using a conventional glucose analyzer in the ICU is clinically acceptable as an alternative calculation of IDVG, although approximated and original IDVGs are not equivalent.

## Abbreviations

AIC = Akaike's information criterion; ICU = intensive care unit; IDVG = initial distribution volume of glucose; SD = standard deviation.

## Competing interests

The author(s) declare that they have no competing interests.

## Authors' contributions

HI designed the study, performed statistical analysis and drafted the manuscript. HN, HO and TT collected data from the patients and performed calculations. KH designed the study and evaluated the data. All authors read and approved the final manuscript.

## Figures and Tables

**Table 1:** Patient demographics

Characteristic/parameter	Value
Number of patients	50
Sex (male/female)	36/14
Age (years)	62 ± 12 (34–79)
Body weight (kg)	59.1 ± 12.9 (37.8–102)
Body surface area (m^2^)	1.60 ± 0.19 (1.27–2.14)
Number of patients receiving mechanical ventilation	26
Number of patients receiving catecholamines^a^	14
Number of patients receiving insulin^b^	7
Number of patients receiving on continuous haemodiafiltration	4

Values are presented as mean ± standard deviation (range) or as number of patients.

^a^Catecholamines: an infusion of dopamine, dobutamine, noradrenaline, or adrenaline.

^b^Insulin: continuous insulin infusion.

**Table 2:** Approximated initial distribution volume of glucose using the incremental glucose level at 3 min postinjection

Δgl 3 min (mg/100 ml)	IDVG (l)	Δgl 3 min (mg/100 ml)	IDVG (l)	Δgl 3 min (mg/100 ml)	IDVG (l)
31	12.3	61	6.6	91	4.3
32	12.0	62	6.5	92	4.2
33	11.8	63	6.4	93	4.2
34	11.5	64	6.3	94	4.2
35	11.2	65	6.2	95	4.1
36	11.0	66	6.1	96	4.1
37	10.7	67	6.0	97	4.0
38	10.5	68	5.9	98	4.0
39	10.3	69	5.8	99	4.0
40	10.0	70	5.7	100	3.9
41	9.8	71	5.6	101	3.9
42	9.6	72	5.5	102	3.8
43	9.4	73	5.4	103	3.8
44	9.2	74	5.4	104	3.8
45	9.0	75	5.3	105	3.7
46	8.8	76	5.2	106	3.7
47	8.7	77	5.1	107	3.7
48	8.5	78	5.1	108	3.7
49	8.3	79	5.0	109	3.6
50	8.1	80	4.9	110	3.6
51	8.0	81	4.8	111	3.6
52	7.8	82	4.8	112	3.5
53	7.7	83	4.7	113	3.5
54	7.5	84	4.7	114	3.5
55	7.4	85	4.6	115	3.5
56	7.2	86	4.5	116	3.5
57	7.1	87	4.5	117	3.4
58	7.0	88	4.4	118	3.4
59	6.9	89	4.4	119	3.4
60	6.7	90	4.3	120	3.4

Each initial distribution volume of glucose (IDVG) was calculated using a formula we previously proposed [9]. Δgl 3 min, increase in glucose concentration above the preinjection level at 3 min after injection.

**Table 3:** Glucose values and approximated initial distribution volume of glucose

Variable	Reference^a^	Plasma^b^	Whole blood^b^
Preinjection glucose (mg/100 ml)	158 ± 42	155 ± 41	153 ± 40
Incremental glucose(mg/100 ml)^c^	58 ± 11	57 ± 12	57 ± 11
Difference in glucose (mg/100 ml)^d^	-	-2 ± 3	-3 ± 4
Approximated IDVG (l)	7.26 ± 1.73	7.38 ± 1.8	7.40 ± 1.65
Difference from original IDVG (l)	-0.17 ± 0.47	-0.05 ± 0.54	-0.04 ± 0.62

Values are presented as mean ± standard deviation. ^a^From plasma glucose values using the same glucose analyzer for original IDVG. ^b^Using a conventional glucose analyzer (combined blood gas and glucose analyzer) for approximated IDVG. ^c^The incremental glucose value at 3 min after glucose injection. ^d^Difference in glucose values in either plasma or whole blood from the reference plasma value. IDVG, initial distribution volume of glucose.

## References

[B1] Ishihara H, Suzuki A, Okawa H, Sakai I, Tsubo T, Matsuki A (2000). The initial distribution volume of glucose rather than indocyanine green derived plasma volume is correlated with cardiac output following major surgery. Intensive Care Med.

[B2] Ishihara H, Matsui A, Muraoka M, Tanabe T, Tsubo T, Matsuki A (2000). Detection of capillary leakage by the indocyanine green and glucose dilutions in septic patients. Crit Care Med.

[B3] Ishihara H, Suzuki A, Okawa H, Ebina T, Tsubo T, Matsuki A (2001). Comparison of the initial distribution volume of glucose and plasma volume in thoracic fluid-accumulated patients. Crit Care Med.

[B4] Ishihara H, Shimodate Y, Koh H, Isozaki K, Tsubo T, Matsuki A (1993). The initial distribution of glucose and cardiac output in the critically ill. Can J Anaesth.

[B5] Nakamura H, Ishihara H, Okawa H, Yatsu Y, Tsubo T, Matsuki A (2005). Initial distribution volume of glucose is correlated with intrathoracic blood volume in hypovolemia and following volume loading in dogs. Eur J Anaesthesiol.

[B6] Gabbanelli V, Pantanetti S, Donati A, Montozzi A, Carbini C, Pelaia P (2004). Initial distribution volume of glucose as noninvasive indicator of cardiac preload: comparison with intrathoracic blood volume. Intensive Care Med.

[B7] Mi W, Ishihara H, Sakai T, Matsuki A (2003). Possible overestimation of indocyanine green-derived plasma volume early after induction of anesthesia with propofol/fentanyl. Anesth Analg.

[B8] Rose BO, Ishihara H, Okawa H, Panning B, Piepenbrock S, Matsuki A (2004). Repeatability of measurements of the initial distribution volume of glucose in haemodynamically stable patients. J Clin Pharm Ther.

[B9] Hirota K, Ishihara H, Tsubo T, Matsuki A (1999). Estimation of the initial distribution volume of glucose by an incremental plasma glucose level at 3 min after i.v. glucose in humans. Br J Clin Pharmacol.

[B10] Akaike H (1974). A new look at the statistical model identification. IEEE Trans Automat Control.

[B11] Ray JG, Hamielec C, Mastracci T (2001). Pilot study of the accuracy of bedside glucometry in the intensive care unit. Crit Care Med.

[B12] Maser RE, Butler MA, Decherney GS (1994). Use of arterial blood with bedside glucose reflectance meters in an intensive care unit: are they accurate?. Crit Care Med.

[B13] Karcher RE, Ingram RL, Kiechle FL, Sykes E (1993). Comparison of the HomoCue berta-glucose photometer and reflotron for open heart surgery. Am J Clin Pathol.

[B14] Kurahashi K, Maruta H, Usuda Y, Ohtsuka M (1997). Influence of blood sample oxygen tension on blood glucose concentration measured using an enzyme-electrode method. Crit Care Med.

[B15] Zöller C, Goetz AE, Weis M, Mörstedt K, Pichler B, Lamm P, Kelger E, Haller M (2001). Continuous cardiac output measurements do not agree with conventional bolus thermodilution cardiac output determination. Can J Anaesth.

[B16] Fogh-Andersen N, Wimberley PD, Thode J, Siggard-Andersen O (1990). Direct reading glucose electrodes detect the molality of glucose in plasma and whole blood. Clin Chim Acta.

[B17] Kuwa K, Nakayama T, Hoshino T, Tominaga M (2001). Relationships of glucose concentrations in capillary whole blood, venous whole blood and venous plasma. Clin Chim Acta.

[B18] Savolainen K, Vitala A, Puhakainen E, Väisänen M (1990). Problems with the use of whole blood as a sample material in novel direct glucose analysers. Scand J Clin Lab Invest.

[B19] Diaz-Parejo P, Stahl N, Xu W, Reinstrup P, Ungerstedt U, Nodstrom CH (2003). Cerebral energy metabolism during transient hyperglycaemia in patients with severe brain trauma. Intensive Care Med.

